# Correction to “TaELP2 Interacts With TaDCL1 and Negatively Regulates Wheat Resistance Against Stripe Rust”

**DOI:** 10.1111/mpp.70262

**Published:** 2026-04-16

Li, M., Y. Chen, S. Guo, et al. 2026. “TaELP2 Interacts With TaDCL1 and Negatively Regulates Wheat Resistance Against Stripe Rust.” *Molecular Plant Pathology* 27: e70225. https://doi.org/10.1111/mpp.70225.

1. Funding Acknowledgment Correction

In the Acknowledgments section of this article, the text “Key R&D Program of Shandong Province (2023LZGC002)” was incorrect.

This should have read: “Key R&D Program of Shandong Province, China (2023LZGC002).”

2 Figure 4 Label Correction

In Figure 4c, the label “GST 空” should be corrected to “GST” by removing the character “空”.

**Figure:**
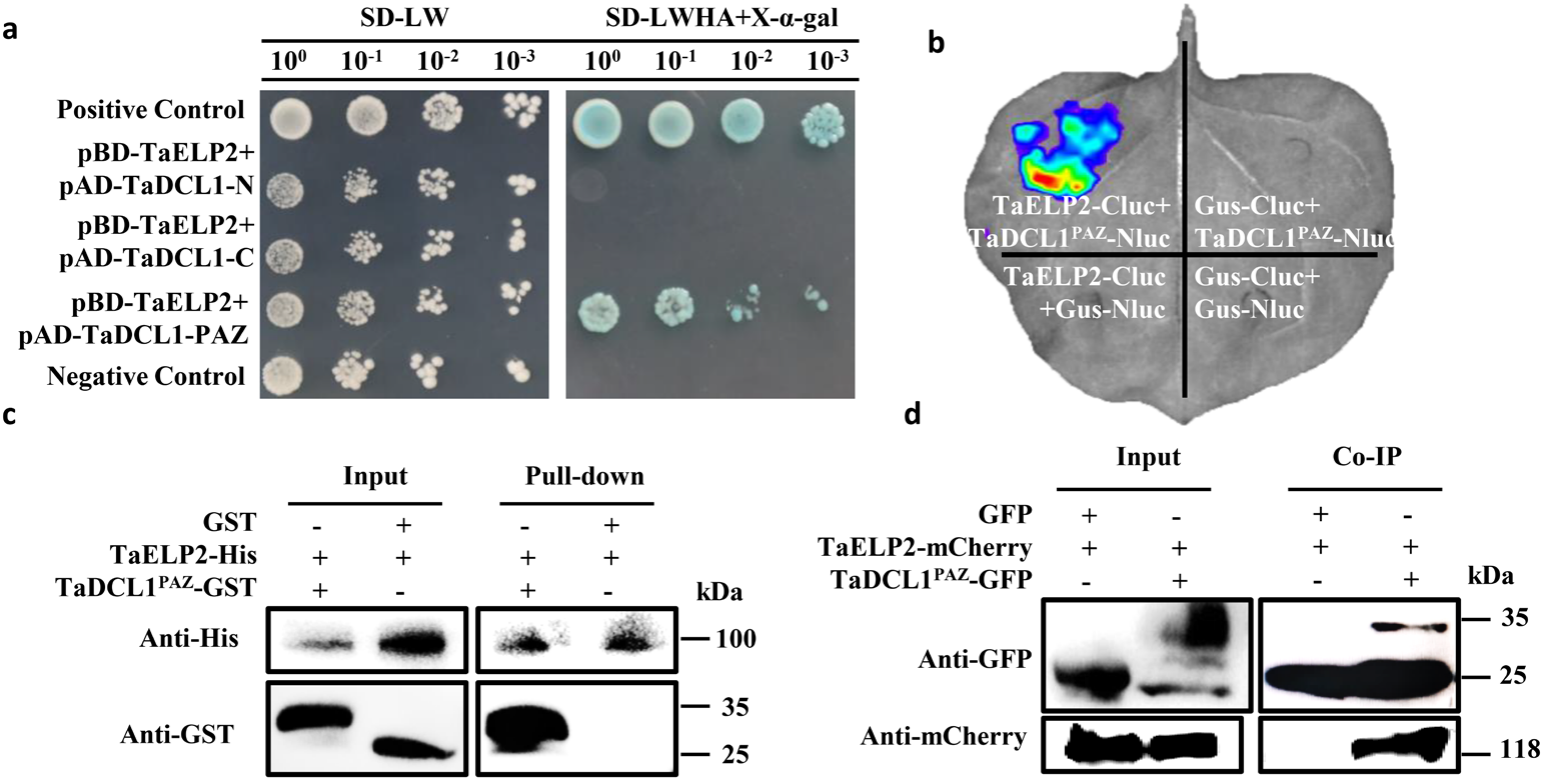
FIGURE 4

3 Statistical Description Correction

In the “4.9 Statistical Analysis” section, the text “Statistical significance was determined by Student's *t*‐test, with *p < 0.05, **p < 0.01, and ns indicating statistically significant.” was incorrect. This should have read: “Statistical significance was determined by Student's *t*‐test, with *p < 0.05, **p < 0.01, and ns indicating no statistically significant difference.”

We apologize for these errors and any inconvenience they may have caused.

